# Cervical cancer screening using DNA methylation triage in a real-world population

**DOI:** 10.1038/s41591-024-03014-6

**Published:** 2024-06-04

**Authors:** Lena Schreiberhuber, James E. Barrett, Jiangrong Wang, Elisa Redl, Chiara Herzog, Charlotte D. Vavourakis, Karin Sundström, Joakim Dillner, Martin Widschwendter

**Affiliations:** 1 European Translational Oncology Prevention and Screening Institute, Hall in Tirol, Austria; 2 Research Institute for Biomedical Aging Research, Universität Innsbruck, Innsbruck, Austria; 3 Center for Cervical Cancer Elimination, Department of Clinical Science, Intervention and Technology, Karolinska Institutet, Stockholm, Sweden; 4 General Hospital Hall, Tirol Kliniken, Hall in Tirol, Austria; 5 Department of Women’s Cancer, UCL EGA Institute for Women’s Health, University College London, London, UK; 6 Department of Women’s and Children’s Health, Karolinska Institutet, Stockholm, Sweden

**Keywords:** Cervical cancer, Risk factors, Predictive markers

## Abstract

Cervical cancer (CC) screening in women comprises human papillomavirus (HPV) testing followed by cytology triage of positive cases. Drawbacks, including cytology’s low reproducibility and requirement for short screening intervals, raise the need for alternative triage methods. Here we used an innovative triage technique, the WID-qCIN test, to assess the DNA methylation of human genes *DPP6*, *RALYL* and *GSX1* in a real-life cohort of 28,017 women aged ≥30 years who attended CC screening in Stockholm between January and March 2017. In the analysis of all 2,377 HPV-positive samples, a combination of WID-qCIN (with a predefined threshold) and HPV16 and/or HPV18 (HPV16/18) detected 93.4% of cervical intraepithelial neoplasia grade 3 and 100% of invasive CCs. The WID-qCIN/HPV16/18 combination predicted 69.4% of incident cervical intraepithelial neoplasia grade 2 or worse compared with 18.2% predicted by cytology. Cytology or WID-qCIN/HPV16/18 triage would require 4.1 and 2.4 colposcopy referrals to detect one cervical intraepithelial neoplasia grade 2 or worse, respectively, during the 6 year period. These findings support the use of WID-qCIN/HPV16/18 as an improved triage strategy for HPV-positive women.

## Abstract (web summary)

In a real-world Swedish cohort of 28,017 women, screening for cervical cancer using a DNA methylation-based tool, the WID-qCIN test, either alone or in combination with HPV16/18 genotyping, showed better performance than cytology in triaging HPV-positive women.

## Main

Cervical cancer (CC) screening is among the most successful strategies for cancer prevention. In combination with high human papillomavirus (HPV) vaccination coverage, cervical screening with substantial uptake is an essential part of the global strategy to eventually eliminate CC^1^. Cytology-based cervical screening requires a complex infrastructure, well-trained workforce and short screening intervals. The proven superiority of testing for oncogenic HPVs (HPV 16, 18, 31, 33, 35, 39, 45, 51, 52, 56, 58, 59, 66 or 68) as an objective and examiner-independent technique with high sensitivity and prolonged protection against cervical intraepithelial neoplasia (CIN; grades 1, 2 or 3) grade 2 or worse (CIN2+)^2–6^ has resulted in international guidelines recommending a transition from primary cytology-based to primary HPV-based screening^7–9^. Due to increased prevalence of HPV in women <30 years of age^10^ leading to low specificity of HPV testing, primary HPV screening is only recommended in women ≥30 years of age. An HPV screen-positive result requires cytology-based triaging so that only HPV- and cytology-positive women are referred for colposcopy and biopsy^7^. In high-income countries, HPV-positive women typically undergo triaging with cytology. In contrast, patients positive for the main oncogenic HPV types (that is, HPV16 and HPV18) are referred directly for colposcopy in the United States^11,12^. At present, most HPV screening tests provide at least partial information on HPV genotypes. Emphasis is increasing to utilize the information on HPV16/18, indicating higher risk for disease progression, in the screening algorithms^11^.

Cytology shows limited and highly variable sensitivity^13^ that has been observed to decrease over time^14^. It requires equipment and expertise that differs from HPV testing, without being applicable for self-samples. The patients who test positive for HPV through self-sampling need to be reinvited for a separate cytology sample, potentially impacting attendance rates adversely. Therefore, improved strategies for triaging HPV-positive women are essential.

The proof of principle strategy to utilize DNA methylation (DNAme) tests on a noncytological sample collected from the cervicovaginal region for detection of cervical (pre)cancer was demonstrated 20 years ago^15^. Since then, several DNAme-based markers have been developed and applied in different settings^16–25^, predominantly representing case–control studies or small cohort sets with fewer than a thousand volunteers. Recently, we developed the WID-qCIN test, assessing DNAme across three regions of the human genes: *DPP6*, *RALYL* and *GSX1*. The latter were selected from an epigenome-wide screen of 850,000 CpGs in 170 CIN3+ cases and 202 controls. A preliminary assay validation was conducted in both a diagnostic (case–control) and a predictive setting (nested case–control) and a total of 761 samples^26^. In this article, we optimized the WID-qCIN test and applied it to HPV-positive women from a real-life population-based cohort of the 28,017 women ≥30 years of age having attended screening in the capital region of Sweden between 1 January and 31 March 2017. We assessed the predictive performance of the WID-qCIN test in combination with HPV16/18 genotyping compared with cytology to triage HPV-positive women.

## Results

### Study population

**Fig. 1:**
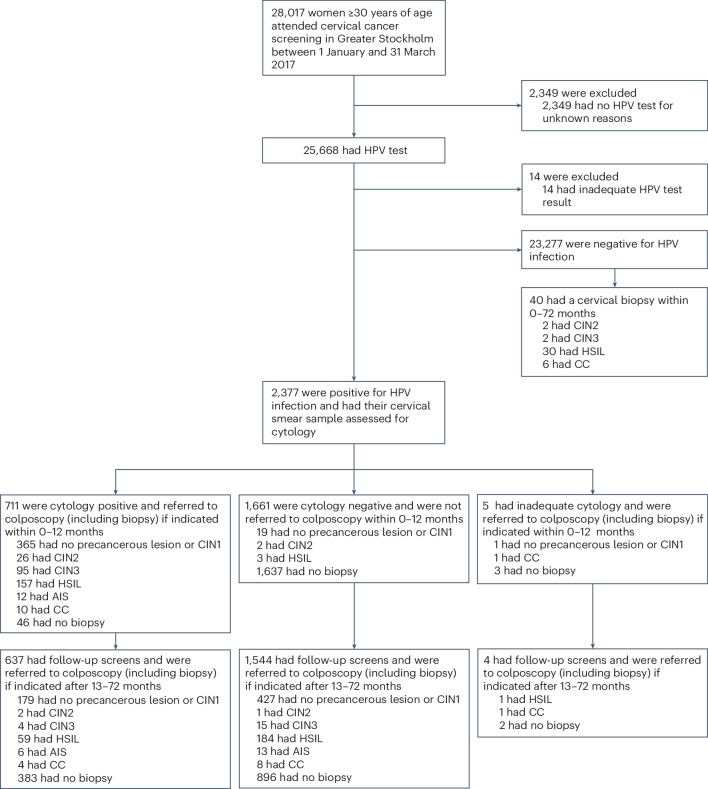
The KI-q1-2017 study population. HSIL is reflective of CIN2 or CIN3.

Between 1 January and 31 March 2017, 28,017 women ≥30 years of age participated in cervical screening in the capital region of Stockholm (the KI-q1-2017 cohort). A total of 2,377 women tested positive for HPV (that is, positive for one or more of the HPV types 16, 18, 31, 33, 35, 39, 45, 51, 52, 56, 58, 59, 66 and 68) and cytology was assessed. Of these, 711 were cytology positive (atypical squamous cells of undetermined significance or worse (ASC-US+)) and were then referred for colposcopy and histological assessment. The biopsies obtained within the first 12 months as a consequence of the baseline screen were defined as prevalent cases (306 CIN2+, of which 11 were CCs) (Fig. 1). Biopsies obtained after 12 months were defined as incident cases (271 CIN2+ of which 11 were CCs; prevalent CIN2+ cases for whom data from follow-up screens or colposcopy were also reported between months 13 and 72 were excluded from the incidence analyses). The average follow-up time of women without CIN2+ was 40.3 months (range from 0.3 to 71.4 months).

The mean age of HPV-positive women was 40.8 years (range 30–64 years); 654 (27.5%) were HPV16 and/or HPV18 (HPV16/18) positive, and 686 (28.9%) were WID-qCIN positive. Among WID-qCIN-positive women, 49.4% and 41.4% were cytology positive and HPV16/18 positive, respectively (Table 1). A total of five women had inadequate cytology results, three were missing HPV subtype information and 90 had inconclusive WID-qCIN results (Supplementary Note 1).

**Table 1:** Characteristics of HPV-positive women of the KI-q1-2017 cohort attending CC screening in Greater Stockholm between 1 January and 31 March 2017

Characteristic	All (*n* = 2,377)	WID-qCIN negative (*n* = 1,601)	WID-qCIN positive (*n* = 686)	WID-qCIN inadequate (*n* = 90)
Age (years)				
Mean (range)	40.8 (30–64)	40.2 (30–64)	42.0 (30–63)	42.6 (30–62)
Cytology result at baseline visit, *n* (%)				
Negative	1,661 (69.9)	1,249 (78.0)	346 (50.4)	66 (73.3)
Positive	711 (29.9)	352 (22.0)	339 (49.4)	20 (22.2)
Inadequate	5 (0.2)	0 (0.0)	1 (0.1)	4 (4.4)
HPV16/18 test result at baseline visit, *n* (%)				
Negative^a^	1,720 (72.4)	1,251 (78.1)	402 (58.6)	67 (74.4)
Positive	654 (27.5)	347 (21.7)	284 (41.4)	23 (25.6)
Inadequate^a^	3 (0.1)	3 (0.2)	0 (0.0)	0 (0.0)
Prevalent (0–12 months)				
Histopathological diagnosis, *n* (%)				
Normal or CIN1	385 (16.2)	263 (16.4)	115 (16.8)	7 (7.8)
CIN2	28 (1.2)	12 (0.7)	16 (2.3)	0 (0.0)
CIN3	95 (4.0)	13 (0.8)	78 (11.4)	4 (4.4)
HSIL	160 (6.7)	38 (2.4)	111 (16.2)	11 (12.2)
AIS	12 (0.5)	3 (0.2)	9 (1.3)	0 (0.0)
CC	11 (0.5)	1 (0.1)	10 (1.5)	0 (0.0)
No biopsy performed	1,686 (70.9)	1,271 (79.4)	347 (50.6)	68 (75.6)
Incident (13–72 months)^b^				
Histopathological diagnosis, *n* (%)				
Normal/CIN1	551 (23.2)	413 (25.8)	124 (18.1)	14 (15.6)
CIN2	3 (0.1)	2 (0.1)	1 (0.1)	0 (0.0)
CIN3	16 (0.7)	4 (0.2)	12 (1.7)	0 (0.0)
HSIL	225 (9.5)	122 (7.6)	92 (13.4)	11 (12.2)
AIS	16 (0.7)	5 (0.3)	11 (1.6)	0 (0.0)
CC	11 (0.5)	6 (0.4)	4 (0.6)	1 (1.1)
No biopsy performed	1,028 (43.2)	814 (50.8)	174 (25.4)	40 (44.4)

^a^HPV16/18 negative or inadequate samples were positive for one or more other high risk HPV strains (HPV31, 33, 35, 39, 45, 51, 52, 56, 58, 59, 66 or 68).

^b^Incident cases cover all histopathological findings excluding prevalent CIN2+ cases and samples without follow-up.

The percentages may not total 100 due to rounding. HSIL is reflective of CIN2 or CIN3. Cytology positive refers to ASC-US+ and cytology negative to negative for intraepithelial lesion or malignancy (NILM).

### Detection of prevalent disease

The sensitivity of cytology to detect prevalent CIN2+ cases defined by histopathology was, by definition, very high at 98.4% since only cytology-positive women in the cohort had been referred for colposcopy and thus histopathological biopsy. Whereas HPV16/18 genotyping detected approximately half of the CIN2+ cases (53.3%, 95% confidence interval (CI) 47.5–58.9), the sensitivity of the stand-alone WID-qCIN testing (77.0%, 95% CI 71.6–81.6) or WID-qCIN testing in combination with HPV16/18 genotyping (further on referred to as WID-qCIN/HPV16/18 (85.9%, 95% CI 81.3–89.6)) was significantly higher (*P* < 0.01 and *P* < 0.01, respectively). Importantly, HPV16/18 alone would have missed >40% of CIN3 cases (sensitivity 58.9%, 95% CI 48.4–68.8), and the sensitivity of the WID-qCIN test alone (85.7%, 95% CI 76.4–91.9) or the WID-qCIN/HPV16/18 (93.4%, 95% CI 85.7–97.3) was significantly higher for CIN3 detection (*P* < 0.01 and *P* < 0.01, respectively). All CCs were detected by HPV16/18 or the WID-qCIN/HPV16/18. The WID-qCIN test detected 10 out of 11 CCs (Table 2).

**Table 2:** Performance of cytology, HPV16/18, WID-qCIN and WID-qCIN/HPV16/18 to detect prevalent disease among HPV-positive women

Parameter	Cytology		HPV16/18		WID-qCIN		WID-qCIN/HPV16/18	
	*n*/total *n*	% (95% CI)	*n*/total *n*	% (95% CI)	*n*/total *n*	% (95% CI)	*n*/total *n*	% (95% CI)
Specificity								
≤CIN1	1,656/2,067	80.1 (78.3–81.8)	1,577/2,068	76.3 (74.4–78.1)	1,534/1,996	76.9 (74.9–78.7)	1,210/1,993	60.7 (58.5–62.9)
Sensitivity								
CIN2+	300/305	98.4 (96.0–99.4)	163/306	53.3 (47.5–58.9)	224/291	77.0 (71.6–81.6)	250/291	85.9 (81.3–89.6)
CIN2	26/28	92.9 (75.0–98.8)	10/28	35.7 (19.3–55.9)	16/28	57.1 (37.4–75.0)	20/28	71.4 (51.1–86.0)
HSIL	157/160	98.1 (94.2–99.5)	76/160	47.5 (39.6–55.5)	111/149	74.5 (66.6–81.1)	124/149	83.2 (76.0–88.7)
CIN3	95/95	100.0 (95.2–100.0)	56/95	58.9 (48.4–68.8)	78/91	85.7 (76.4–91.9)	85/91	93.4 (85.7–97.3)
AIS	12/12	100.0 (69.9–100.0)	10/12	83.3 (50.9–97.1)	9/12	75.0 (42.8–93.3)	10/12	83.3 (50.9–97.1)
CC	10/10	100.0 (65.5–100.0)	11/11	100.0 (67.9–100.0)	10/11	90.9 (57.1–99.5)	11/11	100.0 (67.9–100.0)
PPV								
CIN2+	300/711	42.2 (38.5–45.9)	163/654	24.9 (21.7–28.5)	224/686	32.7 (29.2–36.3)	250/1033	24.2 (21.6–27.0)
NPV								
CIN2+	1,656/1,661	99.7 (99.3–99.9)	1,577/1,720	91.7 (90.3–92.9)	1,534/1,601	95.8 (94.7–96.7)	1,210/1,251	96.7 (95.5–97.6)

The 95% CIs for proportions were computed using the Wilson method. HSIL is reflective of CIN2 or CIN3. For performance assessments, five samples with inadequate cytology, three samples without HPV subtype results and 90 samples with inconclusive WID-qCIN results were excluded from analyses. NPV, negative predictive value; PPV, positive predictive value.

The specificity (≤CIN1) was comparable for cytology (80.1%, 95% CI 78.3–81.8), HPV16/18 (76.3%, 95% CI 74.4–78.1) and WID-qCIN (76.9%, 95% CI 74.9–78.7). The specificity for the WID-qCIN/HPV16/18 was significantly lower (60.7%, 95% CI 58.5–62.9) when compared with either HPV16/18 alone (*P* < 0.01) or the WID-qCIN test alone (*P* < 0.01) (Table 2). All incident cases (diagnosed 13–72 months) were regarded as disease free for the purposes of calculating sensitivity and specificity in the prevalent setting (0–12 months).

The association between the numerical values of the WID-qCIN test (defined as the sum percentage methylation across the three genes) and the histological outcomes further substantiates the ability of the WID-qCIN test to indicate disease progression (Supplementary Note 2, Supplementary Fig. 1 and Supplementary Fig. 2).

### Prediction of incident disease

**Fig. 2:**
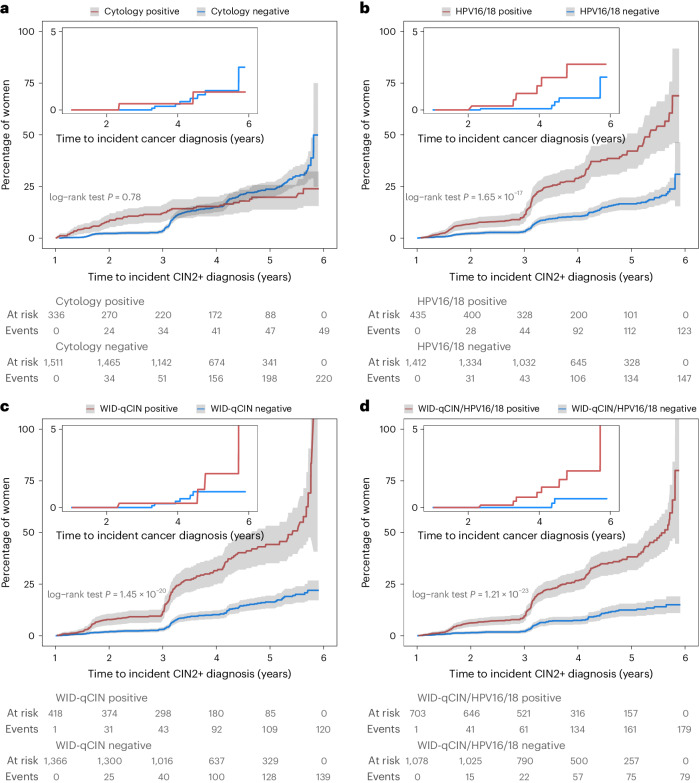
Kaplan–Meier estimates of cumulative incidence rates for incident CIN2+ cases in the KI-q1-2017 cohort. **a**–**d**, Results are stratified according to baseline cytology (**a**), HPV16/18 (**b**), WID-qCIN (**c**) and WID-qCIN/HPV16/18 (**d**). The insets are cumulative incidence curves corresponding to CCs only. The 95% CIs are shown as gray shaded areas. The *P* values assessed using log-rank tests are displayed in light gray within **a**–**d**.

**Extended Data Fig. 1:**
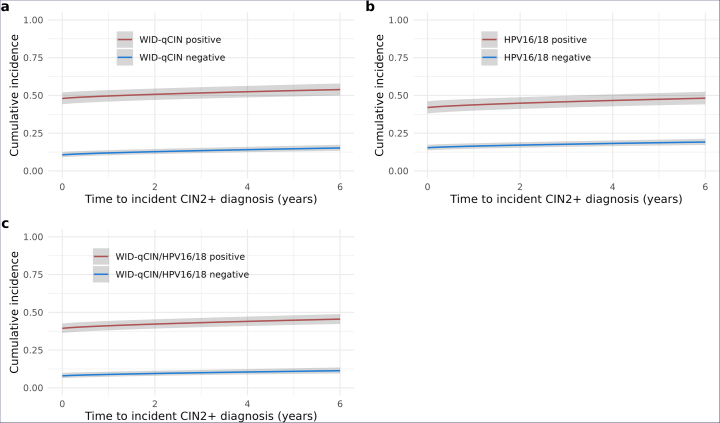
Logistic Weibull mixture model analysis of the KI-q1-2017 cohort. Graphs depict cumulative incidence rates of incident CIN2+ cases stratified according to **a**) WID-qCIN, **b**) HPV16/18, and **c**) WID-qCIN/HPV16/18 analyses. 95% confidence intervals are shown as gray shaded areas. CIN2+ cervical intraepithelial neoplasia or worse, HPV human papillomavirus.

Based on baseline test results, triage with cytology, HPV16/18, the WID-qCIN and the WID-qCIN/HPV16/18 predicted 18.2% (hazard ratio 0.96, 95% CI 0.70–1.31), 45.6% (hazard ratio 2.72, 95% CI 2.14–3.45), 46.3% (hazard ratio 3.01, 95% CI 2.36–3.85) and 69.4% (hazard ratio 3.55, 95% CI 2.73–4.63) of incident CIN2+ cases, respectively (Table 3 and Fig. 2). Whereas cytology only predicted 20.0% of incident CCs (that is, cancers detected more than 12 months after sample collection), HPV16/18 or the WID-qCIN predicted 54.5% and 40.0% respectively. The WID-qCIN/HPV16/18 identified 80.0% of all invasive CCs that developed 13–72 months after the cervical sample collection. The hazard ratios and Kaplan–Meier curves are shown in Table 3 and Fig. 2, respectively. The nonproportional hazards were observed for incident CIN2+ cases stratified according to cytology. This is probably an artifact due to disease detection primarily triggered by a cytology-positive test result. The majority of CIN2+ cases identified because of a (baseline) cytology positive test were detected within 0–12 months (Extended Data Fig. 1), rather than within 13–72 months. Furthermore, the women who were cytology negative at baseline may have tested positive at the second screening round (at approximately 3 years) but are still classified as cytology negative for the purposes of our analysis, which potentially explains why the two curves cross shortly after 3 years.

**Table 3:** Performance of cytology, HPV16/18, WID-qCIN and WID-qCIN/HPV16/18 to predict incident disease among HPV-positive women

Test	All	Incident cases	Hazard ratio (95% CI)	*P* value
	*n*	*n*/total *n* (%)		
CIN2+				
Cytology				
Negative	1,511	220/269 (81.8)	–	
Positive	336	49/269 (18.2)	0.96 (0.70–1.31)	0.78
HPV16/18				
Negative^a^	1,412	147/270 (54.4)	–	
Positive	435	123/270 (45.6)	2.72 (2.14–3.45)	<0.01
WID-qCIN				
Negative	1,366	139/259 (53.7)	–	
Positive	418	120/259 (46.3)	3.01 (2.36–3.85)	<0.01
WID-qCIN/HPV16/18				
Negative	1,078	79/258 (30.6)	–	
Positive	703	179/258 (69.4)	3.55 (2.73–4.63)	<0.01
CC				
Cytology				
Negative	1,511	8/10 (80.0)	–	
Positive	336	2/10 (20.0)	0.93 (0.20–4.40)	0.92
HPV16/18				
Negative^a^	1,412	5/11 (45.5)	–	
Positive	435	6/11 (54.5)	3.87 (1.18–12.68)	0.03
WID-qCIN				
Negative	1,366	6/10 (60.0)	–	
Positive	418	4/10 (40.0)	2.46 (0.69–8.72)	0.16
WID-qCIN/HPV16/18				
Negative	1,078	2/10 (20.0)	–	
Positive	703	8/10 (80.0)	6.44 (1.37–30.35)	0.02

^a^HPV16/18 negative samples were positive for one or more other high-risk HPV strains (that is, HPV31, 33, 35, 39, 45, 51, 52, 56, 58, 59, 66 or 68).

Hazard ratios, 95% CIs and *P* values were calculated using the Cox proportional hazards model. Cytology positive refers to ASC-US+ and cytology negative to negative for intraepithelial lesion or malignancy (NILM).

We also implemented a previously described prevalence–incidence statistical model that considers two features of our cohort^27^. First, it allows for the possibility of undiagnosed prevalent cases (since some incident CIN2+ cases may be prevalent cases that were previously undiagnosed) and, second, the fact that incident cases are interval censored (since disease onset is known only to occur between irregular visits). According to the model, the WID-qCIN test had a hazard ratio of 2.31 (95% CI 1.31–4.08), HPV16/18 had a hazard ratio of 2.47 (95% CI 1.40–4.37) and the WID-qCIN/HPV16/18 had a hazard ratio of 2.83 (95% CI 1.55–5.16) (Supplementary Table 1 and Extended Data Fig. 1). The model failed to converge when the cohort was stratified by cytology, potentially due to poor model fit to the observed nonproportional hazards in cytology-negative and cytology-positive women.

### Cumulative risk by baseline triage test status

Out of 28,017 screened women, 2,377 were HPV positive in the primary screening test. In this HPV-positive subset of women, cytology-based triaging identified 60.8% of CIN2+ cases and 63.6% of CCs over the 72-month study period (Table 4). This required a total of 1,432 colposcopy referrals (split over two screening rounds), resulting in an average of 4.1 colposcopy referrals required to detect one CIN2+ case.

**Table 4:** Estimation of colposcopy referrals in the KI-q1-2017 cohort

Test	CIN2+ detection		CC detection		Colposcopy referrals	Colposcopy referrals required per CIN2+ detection
	*n*/total *n*	% (95% CI)	*n*/total *n*	% (95% CI)	*n*	*n*
Cytology	349/574	60.8 (56.7–64.8)	14/22	63.6 (40.8–82.0)	1,432	4.10
HPV16/18	286/576	49.7 (45.5–53.8)	18/24	75.0 (52.9–89.4)	654	2.29
WID-qCIN	344/550	62.5 (58.3–66.6)	16/23	69.6 (47.0–85.9)	686	1.99
WID-qCIN/HPV16/18	429/549	78.1 (74.4–81.5)	21/23	91.3 (70.5–98.5)	1,033	2.41

The 95% CIs for proportions were computed using the Wilson method.

HPV16/18 triage (based on a single baseline screen) would have detected 49.7% of CIN2+ cases and 75.0% of CCs. Assuming that a positive HPV16/18 result would trigger a colposcopy referral, a total of 654 referrals would be required for this strategy, resulting in an average of 2.3 referrals to detect one CIN2+ case.

WID-qCIN triaging would have detected 62.5% of CIN2+ cases and 69.6% of CCs. A total of 686 referrals would be required, resulting in an average of 2.0 referrals to detect one CIN2+ case.

Finally, WID-qCIN/HPV16/18 triaging would have detected 78.1% of CIN2+ cases and 91.3% of CCs. Importantly, this includes seven out of eight CCs in cytology negative women detected from 13 to 72 months after the baseline screen. A total of 1,033 colposcopy referrals would be required, resulting in an average of 2.4 referrals to detect one CIN2+ case.

## Discussion

We report a large-scale, population-based longitudinal evaluation of triaging HPV-positive women ≥30 years of age participating in cervical screening using an automated molecular test (HPV16/18 in combination with WID-qCIN). The combined approach detects 85.9% (100%) of prevalent and 69.4% (80.0%) of incident CIN2+ (CC) cases. Importantly, the WID-qCIN/HPV16/18 identified seven out of eight cytology-negative women who were subsequently diagnosed with invasive CC, an important finding considering patient outcome. The WID-qCIN/HPV16/18 combination identified almost three times more women with CIN2+ but, nevertheless, resulted in a much lower number of women requiring colposcopy to identify one woman with CIN2+.

The strength of our study lies in the population-based real-life setting, which minimizes biases such as healthy volunteer or greater compliance effects that may be observed with clinical trials. Although only residual samples (that is, after testing for HPV and cytology) were available, 96.2% of samples from all HPV-positive women were suitable for WID-qCIN testing. This is higher compared with data from a study^17^ that assessed the performance of DNAme markers for prevalent disease detection and only included 66.8% of eligible patients that are HPV positive. Despite our unbiased real-life setting, the sensitivity of the WID-qCIN alone to detect prevalent CIN2+ was significantly higher (77.0%, 95% CI 71.6–81.6) than the sensitivity reported in a pooled analysis that assessed the best DNAme markers and excluded highly biased studies (CIN2+ pooled sensitivity was 66.0%, 95% CI 61.0–70.0). The specificity of the WID-qCIN test was also superior (76.9%, 95% CI 74.9–78.7) when compared with other DNAme markers (74.0%, 95% CI 69.0–78.0). Notably, the pooled prevalence of CIN2+ in the published studies was two to three times higher, potentially resulting in an artificial reduction of false-positive cases in these settings^16^.

Whereas no study has assessed the performance of HPV16/18 genotyping in combination with DNAme markers to predict incident and detect prevalent disease, Kremer et al. described a complementary effect of HPV16 and DNAme in predicting the likelihood of CIN2/CIN3 lesions spontaneously regressing, albeit this study was limited by a high proportion (74%) of CIN2 lesions of which only 36% were methylation positive and a low proportion of CIN3 (26%) cases, of which 79% showed methylation^28^. The fact that the WID-qCIN/HPV16/18 detects 93.4% of all prevalent CIN3 cases and predicts 87.5% of all triage cytology-negative invasive CCs indicates that replacing cytology-based triaging by a WID-qCIN/HPV16/18 triage strategy could almost eliminate invasive cancers in an HPV-screened population.

It is important to acknowledge a few limitations of this study. The gradual transition of Swedish pathology laboratories from reporting CIN2–3 lesions both as high-grade squamous intraepithelial lesion (HSIL) limits our analyses of the more severe incident CIN3 cases, equivalent to cervical carcinoma in situ. Furthermore, the ascertainment bias in identifying prevalent cases, where only women with positive cytology results underwent colposcopy with or without biopsy, poses challenges in accurately evaluating the impact of the proposed molecular triage, covering HPV16/18 with WID-qCIN analyses, on prevention of invasive CCs. Ideally, this testing aims to prevent invasive CCs by identifying preinvasive lesions (that is, CIN2, CIN3, adenocarcinoma in situ (AIS) and HSIL), which may be treated before they progress to invasion. Precise assessment of this necessitates the conduction of a prospective randomized clinical trial. Yet, the fact that combined WID-qCIN/HPV16/18 testing predicted all but two cancers developing within 72 months following sample collection indicates that this DNA-based triaging strategy of HPV-positive women has the potential to greatly diminish the number of invasive cancers in the screened population. This further suggests that screening intervals for HPV-positive WID-qCIN/HPV16/18 negative women could be extended to 5 years^29^.

Overall, our data indicate that the DNAme-based WID-qCIN test may complement HPV16/18 genotyping in triaging HPV-positive women with improved performance compared with widely used cytology. The fact that this triage test does not rely on assessment of cellular morphology and can be performed purely on DNA, renders it suitable for screening strategies based on self-sampling. The implementation of WID-qCIN in combination with HPV16/18 screening could help to overcome the issue of resampling patients for triaging after positive HPV results on self-samples.

Cervical screening is an essential pillar of the global strategy to eliminate CC. Furthermore, the World Health Organization advocates for HPV-based screening using self-sampling as a simple strategy that could work also in resource-limited settings. High-performance molecular triaging strategies, such as the WID-qCIN test, which could be readily automated and do not require complex infrastructures, should further facilitate these efforts.

## Methods

### Ethics statement

Ethical approval for the use of samples and linked disease status information in the current study was granted by the Swedish Ethical Review Authority (document number 2014/1242-31/4 and 2022-04693-02) and the Medical University Innsbruck Ethical Committee (reference number 1411/2020). This study was conducted in strict adherence to ethical guidelines and principles.

### Study population

In 2017, the Swedish CC screening program invited all women 23–70 years of age to provide a cervical smear sample every 3–5 years depending on age. The liquid-based cytology specimens were collected by midwives and subjected to HPV testing and cytological assessment^30^. As recommended in the European guidelines for triaging, women aged 30–70 years were primary HPV screened and triaged with cytology upon a positive HPV result. The HPV testing was conducted using the Cobas 4800 platform, which provides genotyping information on HPV16/18 and other oncogenic HPV^31^ strains. All cervical liquid-based cytology samples obtained in Greater Stockholm were biobanked at −25 °C at the Karolinska University Hospital^26,32^. With participation in the nationwide CC screening program, women consented in writing to sample collection and diagnosis, as well as potential sample reuse for future research, as approved by the Swedish Ethical Review Authority. No additional written informed consent was collected from the study participants. Patient consent followed an opt-out principle (that is, samples were biobanked by default unless participant opted out). Due to the screening-based character of the described study, no form of patient compensation was provided.

Women with HPV-positive and cytology-negative results at the baseline visit were invited for follow-up screens within 36 months. Women with HPV-positive and cytology-positive (ASC-US+) results were referred for colposcopy. Upon clinical indication, cervical biopsies were taken and histopathologically assessed. The histopathological findings were reported as CIN2 or CIN3, HSIL, AIS or CC. In 2017, Swedish pathology laboratories gradually replaced reporting of CIN2 and CIN3 separately with HSIL (which includes CIN2 or CIN3 without differentiating between the two). Positive histopathological findings (that is, CIN2+) triggered immediate treatment of the patient according to national guidelines.

All data on screening invitations, HPV, cytologies and histopathological assessments from the cervix are uploaded to the Swedish National Cervical Screening Registry (NKCx) once per year^30^. NKCx was complete up until 31 December 2022. To ensure accurate ascertainment of invasive CC cases, information regarding this disease was obtained from two independent sources. Apart from the NKCx data on cervical histopathologies, we additionally obtained data on invasive CC from the Swedish National Quality Register for Gynecological Cancers (GCR)^33^. For nine women, the NKCx and the GCR disagreed on the diagnosis of invasive cancer. For eight women, the original diagnostic slides and medical charts were reviewed by a pathologist who was unaware of HPV and cytology status. For one woman, the original slides could not be located. In this case, the diagnosis provided by the NKCx was carried forward. The data on race and ethnicity were not collected for this study cohort.

### Study conduct

We conducted a population-based cohort study including all women ≥30 years of age who attended the CC screening program in the capital region of Stockholm between 1 January and 31 March 2017 (the KI-q1-2017 cohort) (Fig. 1). Given the focus on CC screening, only samples from women or those with a cervix were evaluated for this study.

We assessed cervical samples from all HPV-positive women of the KI-q1-2017 cohort with the optimized WID-qCIN test (Supplementary Note 3) and a prespecified threshold (Supplementary Note 4) for what was considered DNAme or WID-qCIN positive. We retrieved information on age, HPV status (negative or positive), HPV16/18 status (for HPV-positive cases only) and cytology outcomes on women having attended the CC screening program between 1 January and 31 March 2017 from NKCx to identify all HPV-positive specimens. Histopathological diagnoses made between 0 and 12 months and between 13 and 72 months were extracted from NKCx, and invasive CC cases were independently verified using data retrieved from the GCR. Furthermore, dates of histopathological diagnosis, last HPV-positive test, last HPV-negative test, last cytology-positive test and last cytology-negative test results between 1 January 2017 and 31 December 2022 were extracted from NKCx.

### Optimized WID-qCIN test

The WID-qCIN, a quantitative real-time PCR test, assesses DNAme in bisulfite-modified DNA in three human gene target regions^26^. The assay has been optimized as described in Supplementary Information (Supplementary Note 3 and Supplementary Fig. 3). Samples of the KI-q1-2017 cohort were analyzed with the optimized and calibrated duplex setup of the WID-qCIN test using a predefined threshold (Supplementary Note 4, Supplementary Table 2 and Supplementary Table 3). The percentage of fully methylated reference (PMR) values were calculated as previously described (Supplementary Note 5)^26^. The samples with SUM−PMR > 0 were defined as WID-qCIN positive and the samples with SUM−PMR = 0 as WID-qCIN negative (Supplementary Fig. 4).

### Statistical analyses

Statistical significance was set to 5%, and 95% CIs were computed for all estimates. The analyses were performed using R (version 4.3.1). The 95% CIs for proportions were computed using the Wilson method in the prop.test function in the stats R package (version 4.3.1). Where applicable, sensitivity or specificity estimates were compared using a two-sided chi-squared test without Yates’ continuity correction using the prop.test function.

Time from sample collection to incident (from 13 to 72 months) CIN2+ (or CC) diagnosis was represented using Kaplan–Meier estimators of cumulative incidence curves using the survfit function in the survival R package (version 3.5–7). The hazard ratios and 95% CIs were calculated using the Cox proportional hazards model using the coxph function in the survival R package (version 3.5–7). The log-rank tests were performed using the survdiff function in the survival R package. The censoring time was defined as the time to the most recent negative test (Supplementary Note 6).

A logistic Weibull mixture model, which considers undiagnosed prevalent disease and interval-censored incident disease, was implemented using the PIMixture R package (version 0.4.4). The odds ratios, hazard ratios and 6-year cumulative incidence estimates, along with 95% CIs, were computed^34^.

### Reporting summary

Further information on research design is available in the Nature Portfolio Reporting Summary linked to this article.

## Online content

Any methods, additional references, Nature Portfolio reporting summaries, source data, extended data, supplementary information, acknowledgements, peer review information; details of author contributions and competing interests; and statements of data and code availability are available at 10.1038/s41591-024-03014-6.

### Supplementary information

Supplementary Information Supplementary Notes 1–6, Figs. 1–4, Tables 1–3 and References.

Reporting Summary

## Data Availability

In consideration of the General Data Protection Regulation by the European Union and the potential risk of patient identification, supplementary analyzed data will not be made publicly available. Specific inquiries requesting additional supplementary data should be directed to M. Widschwendter, MD (Martin.Widschwendter@uibk.ac.at) or J. Dillner, MD, PhD (Joakim.Dillner@ki.se) and will be collaboratively reviewed to ascertain any confidentiality constraints. The evaluation criteria for requests will include overall scientific merit, required anonymization and adherence to data transfer agreements. The response timelines are anticipated to range between 2 and 4 weeks.
