# Supplementary material for: Cervical cancer screening using DNA methylation triage in a real-world population
Source: Nat Med. 2024 Jun 4;30(8):2251–7. doi: 10.1038/s41591-024-03014-6 (PMC11333274; doi:10.1038/s41591-024-03014-6)
Supplement: Supplementary file 1 — Supplementary Notes 1–6, Figs. 1–4, Tables 1–3 and References. [file 41591_2024_3014_MOESM1_ESM.pdf]

nature medicine

Supplementary information https://doi.org/10.1038/s41591-024-03014-6

Cervical cancer screening using DNA methylation triage in a real-world population

In the format provided by the authors and unedited

SUPPLEMENTARY NOTES

Supplementary Note 1: WID-qCIN reproducibility in the KI-q1-2017 cohort

Applying LOB- and LOD-based Cq thresholds following the strategy outlined in the flow-chart (Supplementary Fig. 3), we can report reproducible results for all targets in 2,287 (96.2%) samples. In the 90 (3.8%) samples that failed, 62 (2.6%) were due to insufficient DNA (i.e., COL2A1 Cq values in one or both duplicates >30) and 28 (1.2%) were due to target failure in one or more of the targets.

Supplementary Note 2: WID-qCIN performance in HPV positive women in the prevalence-group of the KI-q1-2017 cohort

Positive correlation between prevalent disease severity and the level of DNAme in WID-qCIN target regions, represented by SUM-PMRs, was observed upon analysis of HPV positive women from the KI-q1-2017 cohort (Supplementary Fig. 1).

Application of the optimized SUM-PMR threshold (SUM-PMR=0) of the WID-qCIN test to discriminate between prevalent ≤CIN1 and CIN2+ cases in the KI-q1-2017 cohort led to an AUC of 0.81 (95% CI: 0.78-0.84) [Supplementary Fig. 2]. Information on assay performance in the complete KI-q1-2017 cohort is provided in detail in the main section of the manuscript.

Supplementary Note 3: WID-qCIN optimization

Following additional optimization measures based on analytical validation guidelines[1], the WID-qCIN test was transformed from a singleplex- to a duplex-based reaction setup allowing improved high-throughput applicability. To assure WID-qCIN reproducibility and to reduce the risk of unspecific target-amplification, limit of detection (LOD)- and limit of blank (LOB)-based Cq thresholds were established for all duplex reactions covering (i) target *RALYL* paired with reference reaction *COL2A1* and (ii) target *GSX1* paired with target *DPP6*. LOB is defined as the analysis of analyte-devoid blanks (i.e., samples containing no target material) resulting in stable target-amplification[2]. LOD describes the lowest concentration of an analyte detected in ≥95% of all tested replicates[1].

To define LOB-based Cq thresholds for *RALYL*, *GSX1* and *DPP6*, nuclease-free H_2O, bisulfite modified unmethylated control DNA (Merck) and 30 randomly selected DNAme negative control samples were assessed in 4-90 replicates depending on sample type. DNA methylation (DNAme) negative control samples were taken from residual samples from the previously published "LBC-CIN Discovery Set" and represented ≤CIN1 controls with low target-specific index CpG methylation according to Illumina MethylationEPIC array results[3]. The LOB-based threshold was defined as the mean (of reproducible) Cq values resulting from blanks and set at 40 for *RALYL*, 34 for *GSX1* and 37 for *DPP6*.

To assess LOD-based Cq thresholds, bisulfite modified methylated control DNA (Zymo) was diluted in bisulfite modified unmethylated control DNA (Merck) at different percentages. All samples were tested in 90 replicates. Target-specific LOD-based thresholds were calculated as the sum of the mean and standard deviation of Cq values resulting from the lowest-concentrated sample detected in ≥95% of all replicates. LOD-based thresholds were set as 36 for *RALYL*, 33 for *GSX1* and 35.5 for *DPP6*.

Supplementary Note 4: WID-qCIN calibration

The calibration set (Supplementary Table 2) consisted of DNA samples from 168 HPV positive and HPV negative clinician-collected liquid-based cytology (LBC) specimens (ThinPrep) randomly selected from the previously reported "LBC-CIN Discovery" and "LBC-CIN Diagnostic" sets[3]. All 168 samples were taken from women ≥23 years of age with histopathologically confirmed CIN2+ or ≤CIN1 and analyzed according to the protocol in Supplementary Fig. 3.

The WID-qCIN SUM-PMR resulted in an AUC of 0.84 (95% CI: 0.77-0.91) [Supplementary Fig. 4] when analyzing the calibration set with 123 ≤CIN1 controls and 45 CIN2+ cases. Based on these observations, the duplex-specific SUM-PMR threshold (SUM-PMR=0) was selected to achieve optimal specificity of 95.9% (95% CI: 90.3-98.5) and clinically significant sensitivity of 71.1% (95% CI: 55.5-83.2) [Supplementary Fig. 4 and Supplementary Table 3]. The SUM-PMR threshold was selected prior to analysis of the KI-q1-2017 cohort.

Supplementary Note 5: WID-qCIN PMR calculation

All samples were assessed in duplicates. Target-PMRs were calculated according to previously published equations[3]. The SUM-PMR was defined as the sum of the three target-PMRs per sample. Samples with *COL2A1* Cq values >30 in one or both replicates were defined as inconclusive and excluded from SUM-PMR calculations. Samples with both replicate target Cq values ≤LOB-based thresholds allowed for target-PMR calculation. Samples with both replicate target Cq values above the LOB-based threshold were defined as having target-PMR=0. Samples with one target Cq value >LOB-based threshold, and a second Cq value that is both ≤LOB-based threshold and >LOD-based threshold were defined as having target-PMR=0. Samples with one target Cq value above the LOB-based threshold and the second Cq value below the LOD-based threshold were defined as inconclusive and were re-tested once (Supplementary Fig. 3). Samples with one or more inconclusive target-PMRs after WID-qCIN re-testing were excluded from SUM-PMR calculations.

Supplementary Note 6: Statistical time-to-event analysis

In the analysis of incident CIN2+ cases there were 271 CIN2+ events and 1,579 censored observations. Time to censoring was defined as the time from sample collection to the most recent negative test. A negative test was defined as HPV negative (n=906), pathology negative (n=353), HPV negative and cytology negative (n=301), or cytology negative in the absence of any HPV results (n=19). A total of 221 samples were removed from the analysis of incident cases (186 with no follow-up data available within 13-72 months and 35 without a negative test with which to define a censoring time).

The logistic Weibull mixture model was fitted using the PIMixture R function with default settings[4]. A total of 306 women were defined as having a prevalent CIN2+ case (confirmed by histopathology results within 0-12 months), 46 were defined as disease free at baseline and having an incident CIN2+ case (disease free status confirmed by histopathology results within 0-12 months and incident CIN2+ case confirmed by histopathology results within 13-72 months), 225 were defined as unknown at baseline and having an incident CIN2+ case (histopathology results unavailable

within 0-12 months and incident CIN2+ case confirmed by histopathology results within 13-72 months), 1,647 were defined as unknown status at baseline and disease free at censor time (histopathology results unavailable within 0-12 months, censoring times defined as above), and 153 were defined as uninformative (118 with no follow-up data available within 0-72 months and 35 without a negative test with which to define a censoring time). Logistic Weibull mixture model results are depicted in Extended Data Fig. 1 and Supplementary Table 1.

SUPPLEMENTARY FIGURES

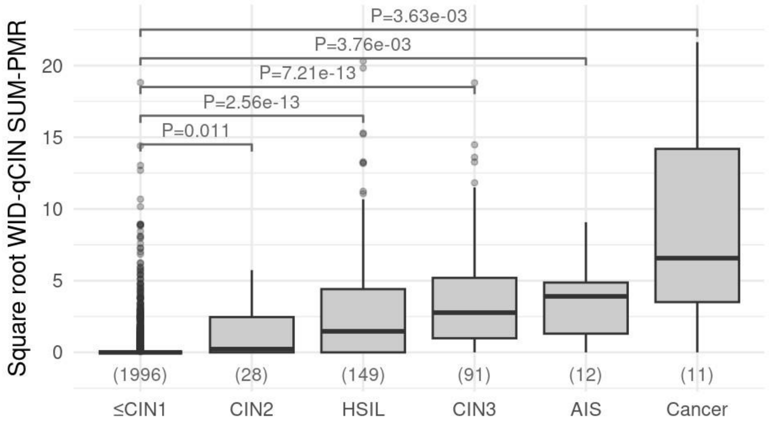

Supplementary Fig. 1: Distribution of square root transformed SUM-PMR values following WID-qCIN based assessment of HPV positive samples from the KI-q1-2017 cohort. Boxplots depict disease-progression-dependent distribution of square root transformed SUM-PMR values. Significant differences between two disease-types were assessed by performing two-sided t-tests. P values indicating the statistical significance of the differences between disease grades are displayed above the respective boxplots in light gray. Each box plot depicts the median value, with boxes and whiskers denoting the interquartile range (IQR). Data points depict outliers falling outside the IQR. Numbers in light gray and brackets underneath the box plots represent biologically independent samples (i.e., n) per disease grade. AIS adenocarcinoma in situ, CIN1/2/3 cervical intraepithelial neoplasia grade 1/2/3, HSIL high grade squamous intraepithelial lesion (reflective of CIN2 or CIN3).

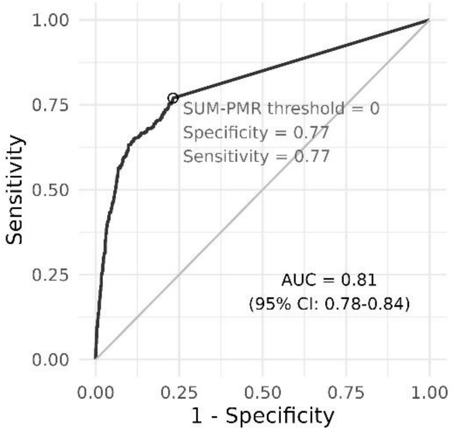

Supplementary Fig. 2: Discrimination between prevalent ≤CIN1 controls and CIN2+ cases of the KI-q1-2017 cohort applying the WID-qCIN. Area under the curve, and 95% confidence intervals were computed using the pROC R package (version 1.18.2). AUC area under the curve.

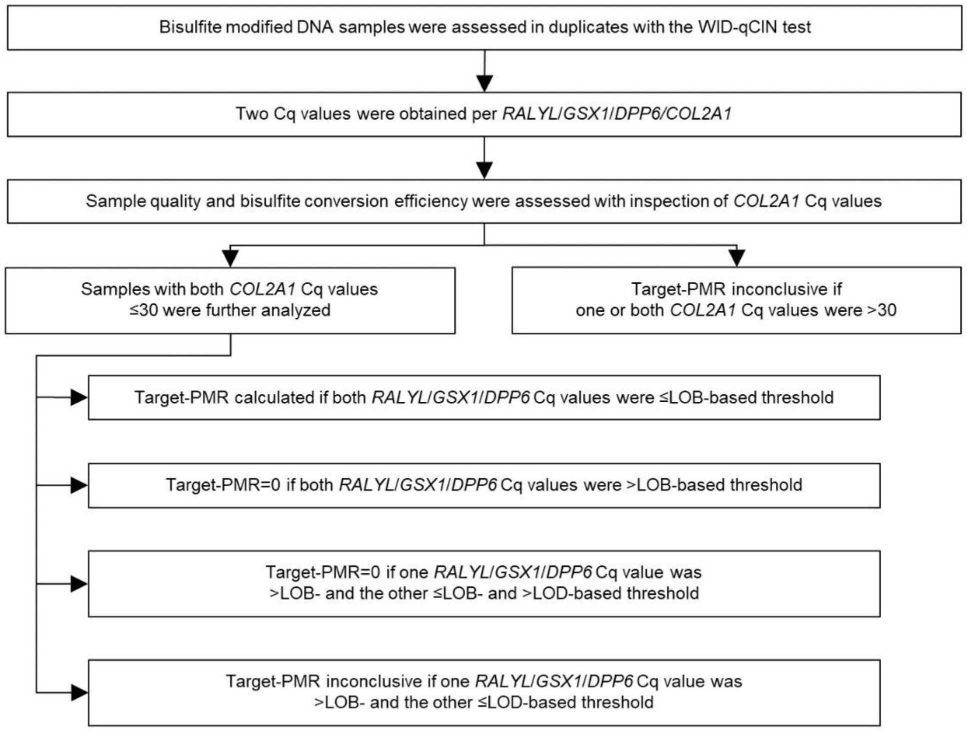

Supplementary Fig. 3: WID-qCIN analysis pathway in the KI-q1-2017 cohort. LOB limit of blank, LOD limit of detection, PMR percentage of fully methylated reference.

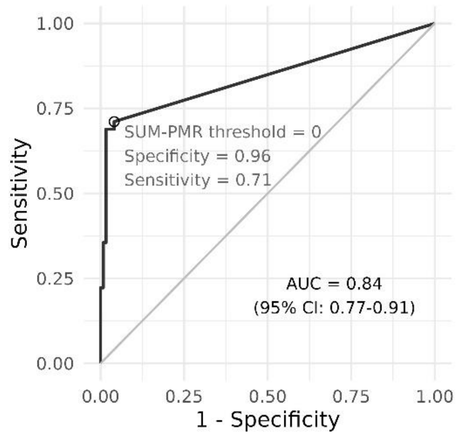

Supplementary Fig. 4: Discrimination between ≤CIN1 controls and CIN2+ cases of the calibration set applying the WID-qCIN test. Area under the curve, and 95% confidence intervals were computed using the pROC R package (version 1.18.2). AUC area under the curve.

SUPPLEMENTARY TABLES

Supplementary Table 1 Cumulative incidence rates of incident CIN2+ in the KI-q1-2017 cohort according to logistic Weibull mixture model analysis. CIN2+ cervical intraepithelial neoplasia grade 2 or worse, HPV human papillomavirus.

Test	Odds ratio (95% CI)	Hazard ratio (95% CI)	CIN2+ cumulative incidence*
**WID-qCIN**			
**Negative**	-	-	0.15 (0.13-0.17)
**Positive**	7.75 (6.06-9.90)	2.31 (1.31-4.08)	0.54 (0.50-0.58)
**HPV16/18**			
**Negative**	-	-	0.19 (0.17-0.21)
**Positive**	3.99 (3.20-4.97)	2.47 (1.40-4.37)	0.48 (0.44-0.52)
**WID-qCIN/HPV16/18**			
**Negative**	-	-	0.11 (0.10-0.14)
**Positive**	7.55 (5.73-9.93)	2.83 (1.55-5.16)	0.46 (0.42-0.49)
*CIN2+ cumulative incidence rates were calculated for a time span of 72 months.			

Supplementary Table 2 Characteristics of the WID-qCIN calibration set. CIN1/2/3+ cervical intraepithelial neoplasia grade 1/2/3 or worse, HPV human papillomavirus.

Characteristic	Calibration set			
	≤CIN1 (n=123)	CIN2+ (n=45)	CIN2 (n=17)	CIN3+ (n=28)
**Age - yr**				
**Mean (Range)**	33.7 (23-59)	33.8 (23-53)	33.9 (23-53)	33.7 (23-51)
**HPV test result - n (%)**				
**Negative**	73 (59.3)	0 (0.0)	0 (0.0)	0 (0.0)
**Positive**	50 (40.7)	45 (100.0)	17 (100.0)	28 (100.0)

Supplementary Table 3 Performance of the WID-qCIN to detect prevalent disease in the calibration set. 95% confidence intervals for proportions were computed using the Wilson method. CIN1/2+ cervical intraepithelial neoplasia grade 1/2 or worse.

Dataset	Specificity		Sensitivity	
	≤CIN1		CIN2+	
	**n/total n**	**% (95% CI)**	**n/total n**	**% (95% CI)**
**Calibration**	118/123	95.9 (90.3-98.5)	32/45	71.1 (55.5-83.2)

SUPPLEMENTARY REFERENCES

1. Bustin SA, Benes V, Garson JA, et al. The MIQE guidelines: minimum information for publication of quantitative real-time PCR experiments. Clin Chem 2009;55(4):611-22. (In eng). DOI: 10.1373/clinchem.2008.112797.
2. Armbruster DA, Pry T. Limit of blank, limit of detection and limit of quantitation. Clin Biochem Rev 2008;29 Suppl 1(Suppl 1):S49-52. (In eng).
3. Herzog C, Sundström K, Jones A, et al. DNA methylation-based detection and prediction of cervical intraepithelial neoplasia grade 3 and invasive cervical cancer with the WID™-qCIN test. Clin Epigenetics 2022;14(1):150. (In eng). DOI: 10.1186/s13148-022-01353-0.
4. Clarke MA, Cheung LC, Castle PE, et al. Five-Year Risk of Cervical Precancer Following p16/Ki-67 Dual-Stain Triage of HPV-Positive Women. JAMA Oncol 2019;5(2):181-186. (In eng). DOI: 10.1001/jamaoncol.2018.4270.
