# Supplementary material for: Cervical cancer screening using DNA methylation triage in a real-world population
Source: Nat Med. 2024 Jun 4;30(8):2251–7. doi: 10.1038/s41591-024-03014-6 (PMC11333274; doi:10.1038/s41591-024-03014-6)
Supplement: Supplementary file 2 — Reporting Summary [file 41591_2024_3014_MOESM2_ESM.pdf]

nature portfolio

Corresponding author(s): Martin Widschwendter

Last updated by author(s): Apr 16, 2024

Reporting Summary

Nature Portfolio wishes to improve the reproducibility of the work that we publish. This form provides structure for consistency and transparency in reporting. For further information on Nature Portfolio policies, see our Editorial Policies and the Editorial Policy Checklist.

Statistics

For all statistical analyses, confirm that the following items are present in the figure legend, table legend, main text, or Methods section.

n/a	Confirmed	
☐	☒	The exact sample size (*n*) for each experimental group/condition, given as a discrete number and unit of measurement
☐	☒	A statement on whether measurements were taken from distinct samples or whether the same sample was measured repeatedly
☐	☒	The statistical test(s) used AND whether they are one- or two-sided<br>*Only common tests should be described solely by name; describe more complex techniques in the Methods section.*
☒	☐	A description of all covariates tested
☐	☒	A description of any assumptions or corrections, such as tests of normality and adjustment for multiple comparisons
☐	☒	A full description of the statistical parameters including central tendency (e.g. means) or other basic estimates (e.g. regression coefficient) AND variation (e.g. standard deviation) or associated estimates of uncertainty (e.g. confidence intervals)
☐	☒	For null hypothesis testing, the test statistic (e.g. *F*, *t*, *r*) with confidence intervals, effect sizes, degrees of freedom and *P* value noted<br>*Give P values as exact values whenever suitable.*
☒	☐	For Bayesian analysis, information on the choice of priors and Markov chain Monte Carlo settings
☐	☒	For hierarchical and complex designs, identification of the appropriate level for tests and full reporting of outcomes
☒	☐	Estimates of effect sizes (e.g. Cohen's *d*, Pearson's *r*), indicating how they were calculated

Our web collection on statistics for biologists contains articles on many of the points above.

Software and code

Policy information about availability of computer code

Data collection	Data were downloaded from the NKCx and the GCR registries and collected using Microsoft Excel.
Data analysis	Statistical significance was set to 5% and 95% confidence intervals (CI) were computed for all estimates. Analyses were performed using R (version 4.3.1). The 95% confidence intervals for proportions were computed using the Wilson method in the prop.test function in the stats R package (version 4.3.1). Where applicable, sensitivity or specificity estimates were compared using a two-sided Chi-squared test without Yates' continuity correction using the prop.test function.<br>Time from sample collection to incident (13 to 72 months) CIN2+ (or cervical cancer) diagnosis was represented using Kaplan-Meier estimators of cumulative incidence curves using the survfit function in the survival R package (version 3.5-7). Hazard ratios (HR) and 95% CIs were calculated using the Cox proportional hazards model using the coxph function in the survival R package (version 3.5-7). Log-rank tests were performed using the survdiff function in the survival R package.<br>A logistic Weibull mixture model, which considers undiagnosed prevalent disease and interval-censored incident disease, was implemented using the PIMixture R package (version 0.4.4). Odds ratios, hazard ratios, and 6-year cumulative incidence estimates, along with 95% confidence intervals were computed.

For manuscripts utilizing custom algorithms or software that are central to the research but not yet described in published literature, software must be made available to editors and reviewers. We strongly encourage code deposition in a community repository (e.g. GitHub). See the Nature Portfolio guidelines for submitting code & software for further information.

Data

Policy information about availability of data

All manuscripts must include a data availability statement. This statement should provide the following information, where applicable:

- Accession codes, unique identifiers, or web links for publicly available datasets
- A description of any restrictions on data availability
- For clinical datasets or third party data, please ensure that the statement adheres to our policy

In consideration of the General Data Protection Regulation (GDPR) by the European Union and the potential risk of patient identification, supplementary analyzed data will not be made publicly available. Specific inquiries requesting additional supplementary data should be directed to Martin Widschwendter, M.D. (Martin.Widschwendter@uibk.ac.at) or Joakim Dillner, M.D., Ph.D. (Joakim.Dillner@ki.se), and will be collaboratively reviewed to ascertain any confidentiality constraints. Evaluation criteria for requests will include overall scientific merit, required anonymization and adherence to data transfer agreements. Response timelines are anticipated to range between two to four weeks.

Research involving human participants, their data, or biological material

Policy information about studies with human participants or human data. See also policy information about sex, gender (identity/presentation), and sexual orientation and race, ethnicity and racism.

Reporting on sex and gender	In this study, only aspects relevant and applicable for individuals with a cervix (i.e., referred to as female or women) were investigated and described.
Reporting on race, ethnicity, or other socially relevant groupings	Data on race and ethnicity were not collected for this study cohort.
Population characteristics	As part of a population-based study, all women attending the cervical cancer screening program in the capital region of Stockholm January 1 and March 31, 2017 who gave consent for sample biobanking and analyses (opt-out system) and were ≥30 years of age, were included, independently of race, ethnicity or socially relevant groupings.
Recruitment	Included were all women ≥30 years of age and having attended the screens within the capital region of Stockholm from January 1 and March 31, 2017 who gave consent for sample biobanking and analyses (opt-out system).
Ethics oversight	The current study was granted by the Karolinska Ethical Committee (Dnr 2014/1242-31/4 and 2022-04693-02) and the Medical University Innsbruck Ethical Committee (Ref# 1411/2020).

Note that full information on the approval of the study protocol must also be provided in the manuscript.

Field-specific reporting

Please select the one below that is the best fit for your research. If you are not sure, read the appropriate sections before making your selection.

☒ Life sciences ☐ Behavioural & social sciences ☐ Ecological, evolutionary & environmental sciences

For a reference copy of the document with all sections, see nature.com/documents/nr-reporting-summary-flat.pdf

Life sciences study design

All studies must disclose on these points even when the disclosure is negative.

Sample size	To conduct a population-based study, all women, ≥30 years of age, attending the cervical cancer screening program in the capital region of Stockholm January 1 and March 31, 2017 who gave consent for sample biobanking and analyses (opt-out system) were included. 28,017 women participated in cervical screening in the capital region of Stockholm and included in the study. Of these, a total of 2,377 women tested positive for HPV and were further assessed for DNA methylation using the WID-qCIN assay. Based on a previous study conducted on biobanked samples (Herzog C, Sundström K, Jones A, Evans I, Barrett JE, Wang J, Redl E, Schreiberhuber L, Costas L, Paytubi S, Dostalek L, Zikan M, Cibula D, Sroczynski G, Siebert U, Dillner J, Widschwendter M. DNA methylation-based detection and prediction of cervical intraepithelial neoplasia grade 3 and invasive cervical cancer with the WID™-qCIN test. Clin Epigenetics. 2022 Nov 21;14(1):150. doi: 10.1186/s13148-022-01353-0. PMID: 36414968; PMCID: PMC9682674) and by using sample size estimates from preceding years according to data of the Swedish National Cervical Screening Registry, a distinct timeframe was defined to supposedly cover sufficient numbers of HPV positive liquid based cytology samples including control, CIN2+, AIS and CC cases.
Data exclusions	Entire cohort was analyzed, no a-priori exclusion.
Replication	To confirm the correctness of the DNA methylation analyses at the EUTOPS lab in Austria, stringent reproducibility guidelines had to be followed when conducting the WID-qCIN assay (see supplementary information regarding LOB- and LOD-based cutoffs). As detailed in the supplementary information, we can report reproducible results for all targets in 2,287 (96.2%) samples. In the 90 (3.8%) samples that failed, 62 (2.6%) were due to insufficient DNA (i.e., COL2A1 Cq values in one or both duplicates >30) and 28 (1.2%) were due to target failure in one or more of the targets. Data obtained from the Swedish cervical cancer screening program could not be retested or replicated and had to be

considered correct. However, for ascertainment of a case of invasive cervical cancer, we required that information about invasive cancer should be obtained from two independent sources. Apart from the NKCx data on cervical histopathologies, we also obtained data in invasive cervical cancer from the Swedish National Quality Register for Gynecological Cancers (GCR) . In cases where the NKCx and the GCR did not agree, the original diagnostic slides and medical charts were reviewed by a pathologist who was unaware of the random allocation and of the HPV and cytology status. For one woman, the original slides could not be located and for this woman we kept the original assignment provided by the NKCx.

Randomization

As part of a population-based study, all women attending the cervical cancer screening program in the capital region of Stockholm January 1 and March 31, 2017 gave consent for sample biobanking and analyses (opt-out system) and were ≥30 years of age, were included, independently of race, ethnicity or socially relevant groupings.

Blinding

Sample collection and biobanking was conducted following the guidelines of the Swedish cervical cancer screening program. HPV positive samples were processed at the EUTOPS lab in Austria in a fully blinded and anonymized manner. Only after completion and confirmation of wetlab analyses, phenotypical information from the Swedish National Cervical Screening Registry was linked to the wetlab results followed by data analyses.

Reporting for specific materials, systems and methods

We require information from authors about some types of materials, experimental systems and methods used in many studies. Here, indicate whether each material, system or method listed is relevant to your study. If you are not sure if a list item applies to your research, read the appropriate section before selecting a response.

Materials & experimental systems

n/a	Involved in the study
☒	☐ Antibodies
☒	☐ Eukaryotic cell lines
☒	☐ Palaeontology and archaeology
☒	☐ Animals and other organisms
☐	☒ Clinical data
☒	☐ Dual use research of concern
☒	☐ Plants

Methods

n/a	Involved in the study
☒	☐ ChIP-seq
☒	☐ Flow cytometry
☒	☐ MRI-based neuroimaging

Clinical data

Policy information about clinical studies
All manuscripts should comply with the ICMJE guidelines for publication of clinical research and a completed CONSORT checklist must be included with all submissions.

Clinical trial registration

Cohort study was not registered.

Study protocol

Cohort study was not registered.

Data collection

To conduct a population-based study, all women, ≥30 years of age, attending the cervical cancer screening program in the capital region of Stockholm January 1 and March 31, 2017 who gave consent for sample biobanking and analyses (opt-out system) were included. 28,017 women participated in cervical screening in the capital region of Stockholm and included in the study. Of these, a total of 2,377 women tested positive for HPV and were further assessed for DNA methylation using the WID-qCIN assay. Data for the whole cohort were obtained from the Swedish National Cervical Screening Registry after ethical approval.

Outcomes

Describe how you pre-defined primary and secondary outcome measures and how you assessed these measures.

Plants

Seed stocks

Report on the source of all seed stocks or other plant material used. If applicable, state the seed stock centre and catalogue number. If plant specimens were collected from the field, describe the collection location, date and sampling procedures.

Novel plant genotypes

Describe the methods by which all novel plant genotypes were produced. This includes those generated by transgenic approaches, gene editing, chemical/radiation-based mutagenesis and hybridization. For transgenic lines, describe the transformation method, the number of independent lines analyzed and the generation upon which experiments were performed. For gene-edited lines, describe the editor used, the endogenous sequence targeted for editing, the targeting guide RNA sequence (if applicable) and how the editor was applied.

Authentication

Describe any authentication procedures for each seed stock used or novel genotype generated. Describe any experiments used to assess the effect of a mutation and, where applicable, how potential secondary effects (e.g. second site T-DNA insertions, mosiacism, off-target gene editing) were examined.
